# Subtle Differences in Brain Architecture in Patients with Congenital Anosmia

**DOI:** 10.1007/s10548-022-00895-z

**Published:** 2022-03-24

**Authors:** Divesh Thaploo, Charalampos Georgiopoulos, Antje Haehner, Thomas Hummel

**Affiliations:** 1 Smell & Taste Clinic, Department of Otorhinolaryngology, TU Dresden, Haus 5, Fetscherstraße 74, 01307 Dresden, Germany; 2 Department of Radiology in Linköping, and Department of Health, Medicine and Caring Sciences, Linköping University, Linköping, Sweden

**Keywords:** Congenital anosmia, Orbitofrontal cortex, Diffusion tensor imaging, Plasticity

## Abstract

People suffering from congenital anosmia show normal brain architecture although they do not have functional sense of smell. Some studies in this regard point to the changes in secondary olfactory cortex, orbitofrontal cortex (OFC), in terms of gray matter volume increase. However, diffusion tensor imaging has not been explored so far. We included 13 congenital anosmia subjects together with 15 controls and looked into various diffusion parameters like FA. Increased FA in bilateral OFC confirms the earlier studies reporting increased gray matter thickness. However, it is quite difficult to interpret FA in terms of gray matter volume. Increased FA has been seen with recovery after traumatic brain injury. Such changes in OFC point to the plastic nature of the brain.

## Introduction

Inability to smell from birth, also known as congenital anomia (CA), is typically associated with absence of olfactory bulb. In a recent study, the authors were astonished by the fact that two congenital anosmic females performed at par with healthy controls in terms of standard olfactory tests despite having no clear olfactory bulbs (Weiss et al. 2020). However, when it comes to odor processing, people with normal or higher sense of smell show a relationship between olfactory performance, measured by odor threshold, identification and discrimination and orbitofrontal cortex (OFC) which is highly significant in the formation of olfactory precepts (Seubert et al. 2013). Using regression models, the authors concluded that gray matter differences in OFC were responsible for variances in odor discrimination but little variances in threshold scores. In CA an increase in gray matter thickness was found to be present bilaterally in OFC and the authors concluded that it may be due to lack of synaptic pruning due to absence of peripheral sensory input (Frasnelli et al. 2013). A recent review related to brain structural changes in congenital or acquired anosmia also indicated an increased gray matter thickness within the OFC in CA while it was reduced in acquired anosmia (Manan et al. 2022). As indicate above, this increase of the gray matter in CA may be explained by the lack of input to OFC from primary olfactory areas in CA, changing the input-dependent development of the brain architecture.

To date, no study has focused on individuals with CA in terms of diffusion tensor imaging (DTI). DTI is a robust tool to investigate structural integrity where one of the measures is fractional anisotropy (FA). Higher FA values indicate more axon myelination (Osuka et al. 2012). FA has been found to be a marker of improved function in various neurodegenerative diseases and recovery from traumatic brain injury (Alba-Ferrara and de Erausquin 2013; Wallace et al. 2018). Increased cerebral myelination has been associated with increased gray matter thickness and FA, both sharing a linear correlation (Kochunov et al. 2011). However, the effect has yet not been clearly understood. The main purpose of the study was to investigate whether FA can explain the differences noted previously in OFC in CA and compare them with healthy controls.

## Methods

We present an investigation in 13 CA participants and 15 healthy controls using a ROI-based approach. Diffusion tensor imaging was performed using 3 T MRI scanner (Verio; Siemens Healthineers, Erlangen, Germany). An eight-channel receiver head coil was used for image data acquisition. DTI was acquired as 2D fast spin echo planar imaging with following specifications; TR = 71 ms, TE = 6 ms, Slice thickness = 2 mm, FoV = 110 × 110, repetitions = 1, flip angle = 180°. Diffusion scans were acquired at b = 0 and b = 800 with number of diffusion directions = 20. Following written informed consent, participants underwent olfactory testing with the Sniffin’ Sticks battery (odor threshold, discrimination, and identification: TDI score) (Oleszkiewicz et al. 2019). Masks for piriform cortex and orbitofrontal cortex (OFC) were adapted and thresholded from two published studies (Fjaeldstad et al. 2017; Seubert et al. 2013) using FSL edit mode (FMRIB software library v6.0.2) (Jenkinson et al. 2012) to include white matter areas and manual removal of any underline gray matter, if required. These ROIs were visually inspected by expert neuroradiologists, who also helped in normalisation and outline of the ROIs. We also analysed the FA values in piriform cortex (PFC) using the same approach. Voxelwise statistical analysis of the FA data was carried out using TBSS (Tract-Based Spatial Statistics (Smith et al. 2006)), part of FSL. TBSS projects all subjects' FA data onto a mean FA tract skeleton, before applying voxelwise cross-subject statistics. Statistical analysis was carried out using SPSSv27 (Armonk, NY, USA: IBM Corp). We used Mann–Whitney U test, a non-parametric test given the sample of the study, where r < 0.3 represents a small effect, r between 0.3 and 0.5 medium effect and r > 0.5 a large effect (r = z/√n; z: standardised test statistic, n: number of samples).

## Results

**Fig. 1:**
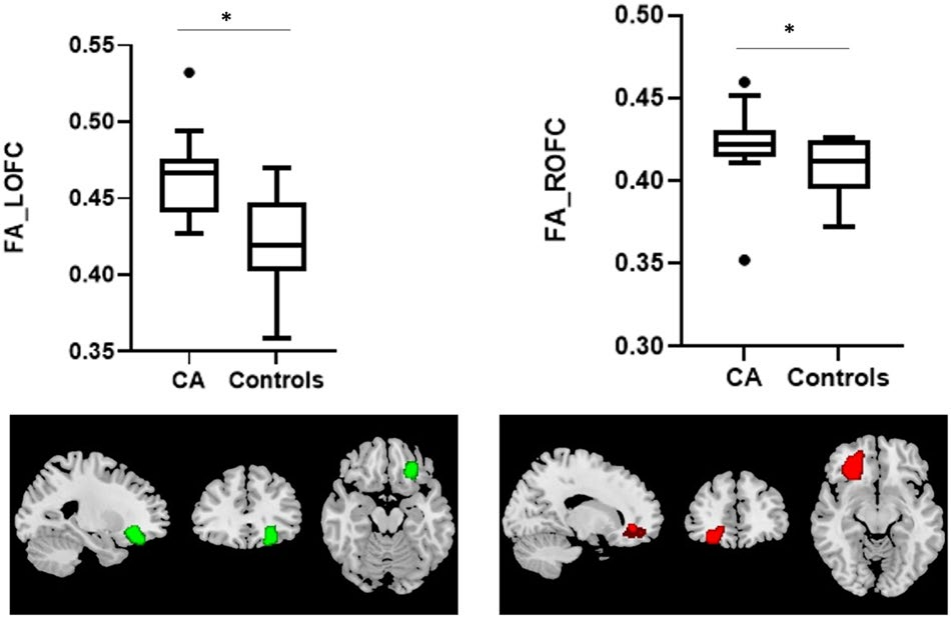
The box plots for FA values in LOFC and ROFC. * denotes significant difference between two groups (p < 0.05). *LOFC* left orbitofrontal cortex, *ROFC* right orbitofrontal cortex, *CA* congenital anosmia subjects. Green and red colour provide the location of left and right orbitofrontal cortex, respectively in standard space

Thirteen CA subjects (mean age 30.6 ± 12.4 years) and 15 healthy controls (38.6 ± 11.3 years) were included in the study. The distribution of age was similar across the groups which was revealed by independent samples Mann–Whitney U test (r = 0.36, p = 0.052). On testing, CA subjects had a significantly lower TDI score (r = 0.85, p = 0.001) (12.69 ± 2.9) as compared to healthy controls (34.1 ± 3.0). Mann–Whitney U test revealed significant changes in FA values within the OFC in each hemisphere between the two groups. FA values within left and right OFC were higher (r = 0.58, p = 0.002 and r = 0.44, p = 0.019, respectively) in CA group (FA in left OFC, 0.49 ± 0.02, right OFC, 0.44 ± 0.01) as compared to healthy controls (FA in left OFC, 0.44 ± 0.01, right OFC, 0.39 ± 0.01) A graphical representation can be seen in Fig. 1. As pertaining to the analysis for PFC, we did not find significant differences between the groups (r = 0.20, p = 0.37 for left PFC and r = 0.17, p = 0.38 for right PFC).

## Discussion

FA values in bilateral OFC were significantly higher in CA as compared to healthy controls. The results of the present study partly confirms an earlier study, where the authors reported higher cortical thickness bilaterally within OFC in CA subjects in terms of an increase in gray matter thickness (Frasnelli et al. 2013). Higher FA values and the increase in cortical thickness within the OFC, which is a secondary olfactory area, suggests the plastic nature of the brain (Sakai 2020). However, the exact implication of FA for gray matter thickness is still unknown. Nonetheless, some studies have observed that increased grey matter volume and higher FA may be related to neuroplasticity (Hsin et al. 2017). However, when we look into the FA values between groups, the differences may be subtle but, nonetheless, they are statistically significant. Some studies have shown absence of differences between both groups with no morphological alterations in primary olfactory cortex (Peter et al. 2020). The authors concluded that the lack of lifelong olfactory experience had no major effect on the primary olfactory cortex. However, there were some changes in OFC which may be the result of developmental processes and also due to the multimodal nature of the OFC. Also, no gray matter alterations in primary olfactory cortex, which includes the piriform cortex, have been seen in rodents. There, postnatal removal of olfactory bulb, severing inputs to primary olfactory cortex, produced little or no alterations in the thickness of the piriform cortex (Friedman and Price 1986; Westrum and Bakay 1986). A study by (Karstensen et al. 2018) on CA patients points to the loss of grey matter volume in medial OFC. However, inclusion of hyposmic patients in the CA group by the authors, could be responsible for such reduced volume in medial OFC as was observed by Yao and colleagues, where patients with hyposmia show atrophy in right orbitofrontal cortex (Yao et al. 2018). Based on the existing literature, and the present findings, we conclude that people with CA have higher FA values in OFC pointing towards the neuroplastic nature of the brain.

## Conclusion

In congenital anosmia the increased FA in OFC and no changes in piriform cortex points to the plastic nature of the brain.

## Data Availability

Not applicable.
